# Chromosome-length genome assembly of *Teladorsagia circumcincta* – a globally important helminth parasite in livestock

**DOI:** 10.1186/s12864-023-09172-0

**Published:** 2023-02-15

**Authors:** Shamshad Ul Hassan, Eng Guan Chua, Erwin A. Paz, Chin Yen Tay, Johan C. Greeff, Dieter G. Palmer, Olga Dudchenko, Erez Lieberman Aiden, Graeme B. Martin, Parwinder Kaur

**Affiliations:** 1 UWA School of Agriculture and Environment, The University of Western Australia, 6009 Crawley, WA Australia; 2 Helicobacter Research Laboratory, The Marshall Centre for Infectious Disease Research and Training, School of Biomedical Sciences, University of Western Australia, Perth, WA Australia; 3 Department of Primary Industries and Regional Development, Western Australia 3 Baron Hay Court, South Perth, 6151 WA Australia; 4 The Center for Genome Architecture, Department of Molecular and Human Genetics, Baylor College of Medicine, 77030 Houston, TX USA; 5 Center for Theoretical Biological Physics, Rice University, 77005 Houston, TX USA; 6 Shanghai Institute for Advanced Immunochemical Studies, ShanghaiTech, Pudong China; 7 Broad Institute of MIT and Harvard, Cambridge, MA USA

**Keywords:** Genome assembly, Brown stomach worm, *Teladorsagia circumcincta*, Hi-C sequencing, 3D-DNA, Bioinformatics

## Abstract

**Background:**

Gastrointestinal (GIT) helminthiasis is a global problem that affects livestock health, especially in small ruminants. One of the major helminth parasites of sheep and goats, *Teladorsagia circumcincta*, infects the abomasum and causes production losses, reductions in weight gain, diarrhoea and, in some cases, death in young animals. Control strategies have relied heavily on the use of anthelmintic medication but, unfortunately, *T. circumcincta* has developed resistance, as have many helminths. Vaccination offers a sustainable and practical solution, but there is no commercially available vaccine to prevent Teladorsagiosis. The discovery of new strategies for controlling *T. circumcincta*, such as novel vaccine targets and drug candidates, would be greatly accelerated by the availability of better quality, chromosome-length, genome assembly because it would allow the identification of key genetic determinants of the pathophysiology of infection and host-parasite interaction. The available draft genome assembly of *T. circumcincta* (GCA_002352805.1) is highly fragmented and thus impedes large-scale investigations of population and functional genomics.

**Results:**

We have constructed a high-quality reference genome, with chromosome-length scaffolds, by purging alternative haplotypes from the existing draft genome assembly and scaffolding the result using chromosome conformation, capture-based, in situ Hi-C technique. The improved (Hi-C) assembly resulted in six chromosome-length scaffolds with length ranging from 66.6 Mbp to 49.6 Mbp, 35% fewer sequences and reduction in size. Substantial improvements were also achieved in both the values for N50 (57.1 Mbp) and L50 (5 Mbp). A higher and comparable level of genome and proteome completeness was achieved for Hi-C assembly on BUSCO parameters. The Hi-C assembly had a greater synteny and number of orthologs with a closely related nematode, *Haemonchus contortus.*

**Conclusion:**

This improved genomic resource is suitable as a foundation for the identification of potential targets for vaccine and drug development.

**Supplementary Information:**

The online version contains supplementary material available at 10.1186/s12864-023-09172-0.

## Background

Roundworms (phylum Platyhelminthes; class Nematoda) include some economically important species that infect livestock globally and incur huge annual losses in production [1, 2]. For example, *Teladorsagia circumcincta*, also known as the brown stomach worm, infects small ruminants including sheep [3] and is one of the major problematic helminth species in the southwestern part of Australia. This region has a Mediterranean-type climate with winter rainfall that favours the propagation of the larval stages of *T. circumcincta* on pasture [4].

The life cycle of *T. circumcincta* continues when third-stage (L_3_) larvae on pasture are ingested by grazing sheep, exsheath and invade the mucosa of the abomasum where they develop into the fourth stage (L_4_). Immature worms emerge from the mucosa into the gastric lumen where they develop into adult males and females and become sexually mature. The infection leads to functional disruption of the gastric mucosa, oedema of abomasal folds and sloughing of the mucosal lining, resulting in increased production of mucus, decreased production of acid, increased serum levels of pepsinogen and, possibly, protein deficiency (hypoalbuminemia). The host can suffer anorexia, dehydration, weight loss and diarrhoea, collectively leading to significant economic losses [2]. The helminth eggs leave the host in faecal material to re-contaminate the pasture and complete the life cycle, thus leading to recurrent infections [1].

For decades, control of the infection has relied on the extensive use of anthelmintic medications that were originally able to control the helminths, including *T. circumcincta.* Unfortunately, this practice has led to widespread development of resistance to some of the most effective anthelmintics on the market, including monepantel [5, 6]. Among the alternative, sustainable options are vaccination, but for *T. circumcincta*, a vaccine is not commercially available [7]. All issues considered; therefore, we need to be able to identify new targets for vaccine and drug development and elucidate the mechanisms that lead to anthelmintic resistance. Clearly, a good starting point in this quest would be a high-quality reference genome assembly.

Advances in high-throughput sequencing technologies over the past two decades have triggered a massive output of genomic data. The improvements in the technology provide an opportunity to revisit the original sequencing and genome assembling attempts. The original sequencing attempt that resulted in a highly fragmented genome thus offers a real opportunity to develop a high-quality genomic resource for *T. circumcincta*, potentially allowing major gains in our basic understanding of the physiology, evolutionary biology, pathogenesis of infection, host immune response, and the mechanisms that underpin the anthelmintic resistance [8, 9].

In the present study, we aimed to improve the current *T. circumcincta* draft genome to a chromosome-length assembly, using chromosome conformation capture technique, or in situ Hi-C [10], and thus increase the value of the genome resource by annotating and analysing it for genome-wide synteny and orthologs.

## Results

### Genome contiguity and completeness

**Fig. 1:**
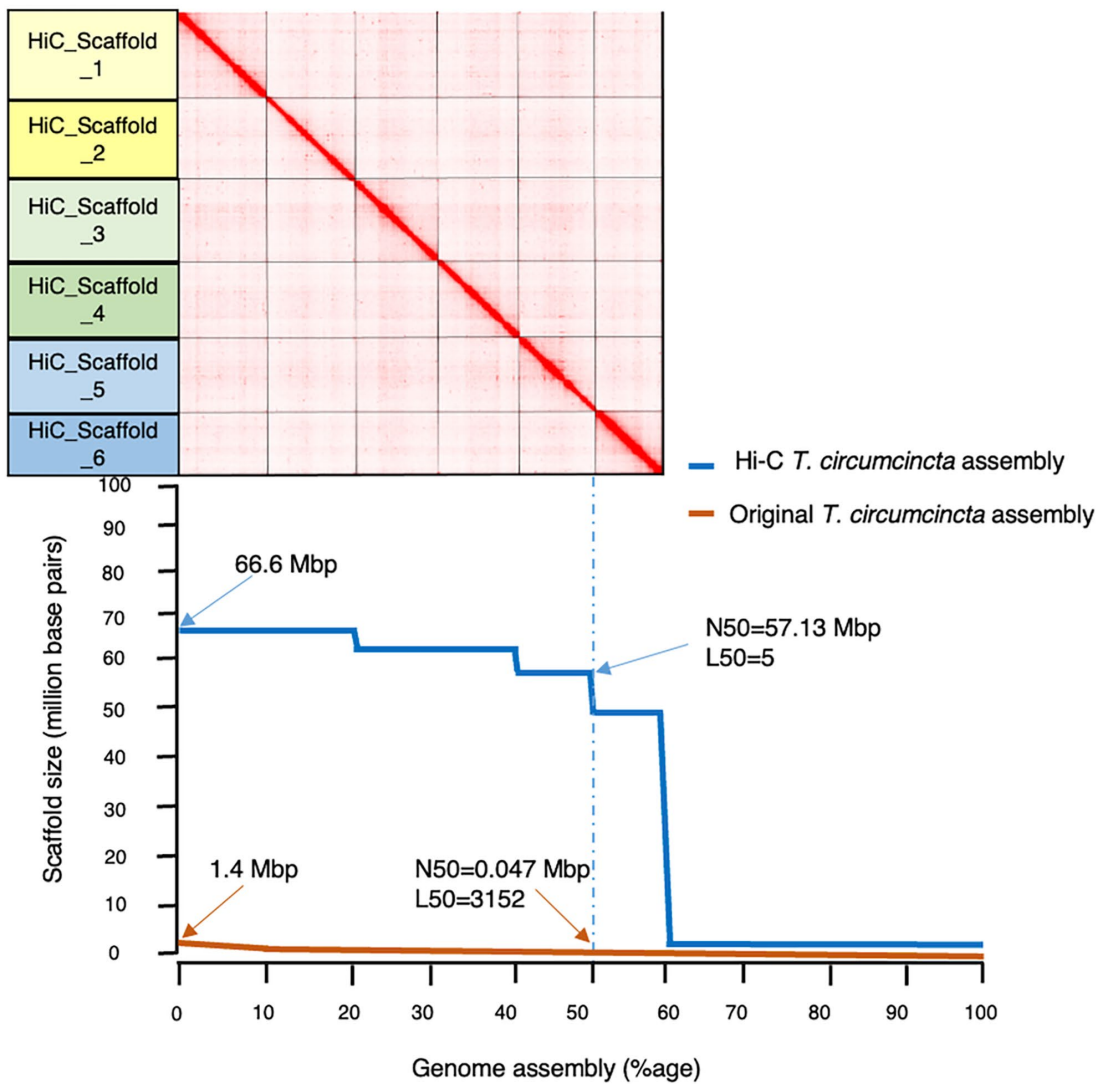
Comparison of the Hi-C and draft genome assemblies for genome contiguity and completeness. Top: Hi-C matrix of the spatial clustering of Hi-C reads to six chromosome-length scaffolds in Hi-C assembly. The interactive contact map is available at https://www.dnazoo.org/assemblies/Teladorsagia_circumcincta. Bottom: comparison of the scaffold lengths of Hi-C and draft genome assemblies (values for N50 and L50 are indicated for both assemblies)

The original draft genome assembly (GCA_002352805.1) was highly fragmented with 81,730 scaffolds, with N50 of 47,089 bp and L50 of 3152, and a total size of 700 Mbp (Table 1). Following the purging of alternative haplotypes and the integration of Hi-C sequencing data, the new Hi-C assembly contained 52,860 scaffolds approximately 35% fewer sequences than the original draft. Notably, of these, six were chromosome-length as shown in Fig. 1, with lengths ranging from 66.6 Mbp to 49.6 Mbp. Substantial improvements were achieved in both the values for N50 (57.1 Mbp) and L50 (5 Mbp). The longest scaffold had increased markedly in length, from approximately 1.4 Mbp in the original assembly to nearly 66.6 Mbp in the Hi-C assembly, while the estimated genome size was reduced from 700 Mbp to 614 Mbp, probably due to improved identification and separation of haplotypes.

**Table 1:** Quality assessments of the original and Hi-C integrated genome assemblies of *T. circumcincta*

Parameters	Original assembly	Purged Hi-C assembly^a^	Chromosomes only^b^
Number of scaffolds	81,730	52,860	6
Total size of scaffolds (bp)	700,607,159	614,147,289	363,201,533
Longest scaffold (bp)	1,473,920	66,586,561	66,586,561
N50 scaffold length (bp) count	47,089	57,133,369	62,733,602
L50 scaffold count	3152	5	3
Number of contigs	213,313	175,861	76,733
Longest contig (bp)	98,345	98,345	98,345
N50 contig length (bp)	3624	4009	5700
L50 contig count	40,623	32,178	14,421
**BUSCO assessment** ^**c**^			
Complete (single-copy + duplicated)	2099 (67%)	2112 (67.5%)	1840 (58.8%)
Complete and single-copy	1835 (58.6%)	1978 (63.2%)	1821 (58.2%)
Complete and duplicated	264 (8.4%)	134 (4.3%)	19 (0.6%)
Fragmented	350 (11.2%)	358 (11.4%)	269 (8.6%)
Missing	682 (21.8%)	661 (21.1%)	1022 (32.6%)

^**a**^Includes all available scaffolds; ^**b**^Includes only six chromosome-length scaffolds; ^**c**^BUSCO assessment was performed using the nematode odb10 dataset which contains 3131 orthologs.

Next, BUSCO (with nematode odb10 data) was used to assess and compare the genome completeness levels of both assemblies. After adding scaffolds (n = 353) to the Hi-C assembly from the draft assembly that contained missing BUSCOs, we detected a higher level of genome completeness in the Hi-C assembly, with 67.5% (2112/3131) of BUSCO genes identified compared to 67% (2099/3131) in the original assembly (Table 1). More importantly, the Hi-C assembly contained 143 more single-copy and 130 fewer duplicated BUSCO genes, than the original assembly, indicating a significant reduction in the number of duplicated sequences. We then examined the genome completeness of only the six chromosome-length scaffolds, achieving an overall completeness score of 58.8% compared to 67.5% in the entire Hi-C-assembly. The sequences for the missing BUSCOs were retrieved manually from https://www.orthodb.org/ and 1269 scaffolds containing missing BUSCOs were added to the six chromosome-length scaffolds and the completeness score rose to 67.1%, very similar to the Hi-C assembly containing 52,860 scaffolds.

### Genome and functional annotations

The genome annotation results generated from the Braker2 pipeline are outlined in Table 2. The annotated Hi-C assembly had fewer genes (28,082) and mRNA transcripts (30,055), compared to the original draft (37,276 genes; 39,896 mRNA transcripts), but the BUSCO assessment scores of both protein sequence sets were highly comparable. In the Hi-C assembly, the overall genome completeness level was 76.7%, slightly less than that of the original assembly (76.9%). However, it is important to note that, in comparison to the original assembly, the Hi-C assembly contained more single-copy (58% vs. 58.6%), fewer duplicates (18.9% vs. 18.1%) and fewer fragmented (8% vs. 7.6%) orthologs, demonstrating the improvement in genome accuracy and fragmentation.

**Table 2:** Comparison of genome annotations in the purged, Hi-C integrated and original genome assemblies of *T. circumcincta*

Parameters	Purged Hi-C assembly	Original assembly
mRNAs (n)	30,055	39,896
Genes (n)	28,082	37,276
Exons (n)	239,113	281,766
CDS (n)	239,106	281,759
Introns (n)	197,880	231,167
Start Codon (n)	26,422	32,685
Stop codon (n)	27,090	33,400
BUSCO assessment*		
Complete (single-copy + duplicated)	2402 (76.7%)	2409 (76.9%)
Complete and single-copy	1834 (58.6%)	1816 (58.0%)
Complete and duplicated	568 (18.1%)	593 (18.9%)
Fragmented	239 (7.6%)	250 (8%)
Missing	490 (15.7%)	472 (15.1%)

*BUSCO assessment was performed using the nematode odb10 dataset which contains 3131 orthologs.

**Fig. 2:**
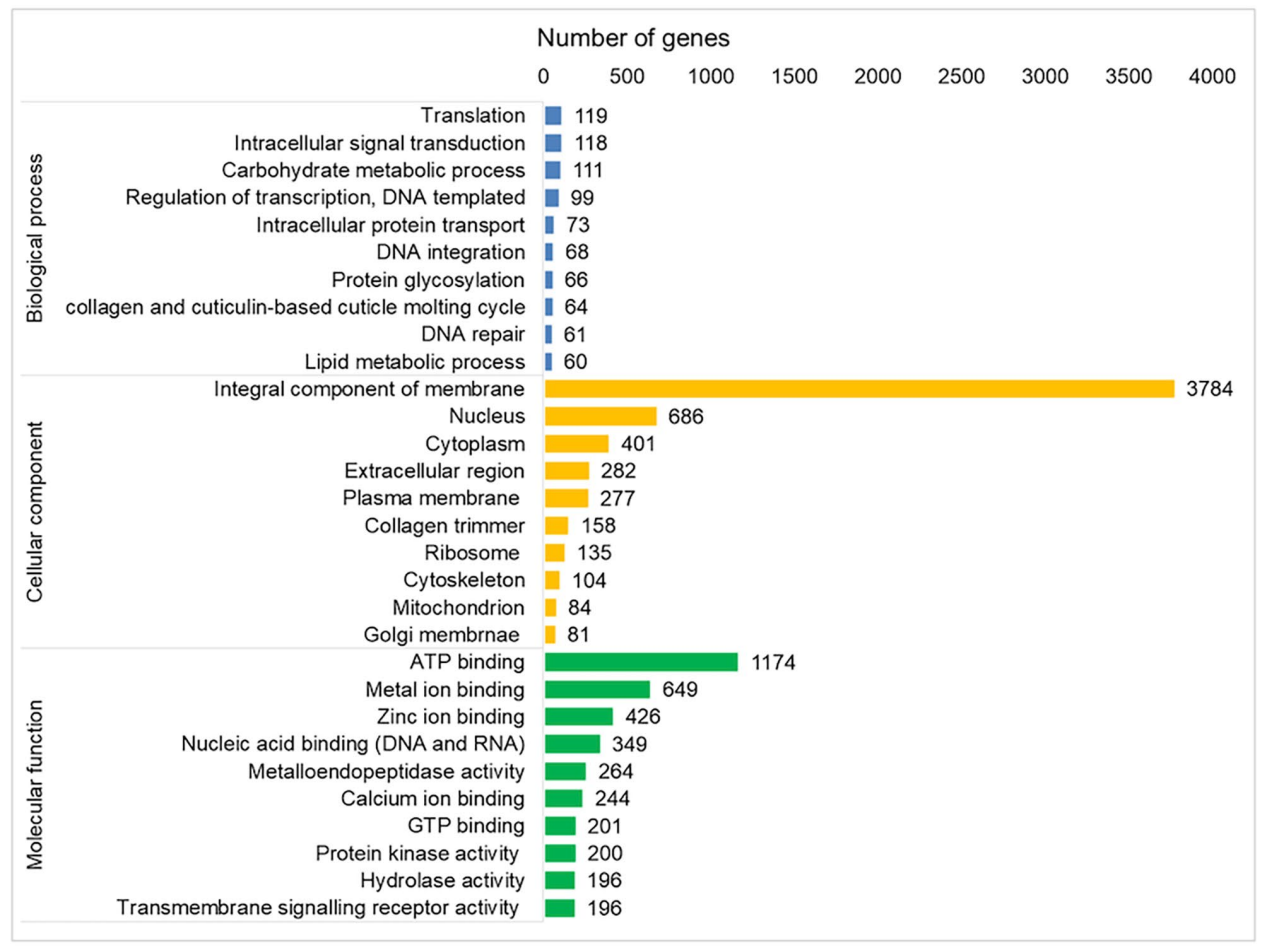
Bar plots depicting the 10 most abundant Gene Ontology (GO) terms in the Hi-C assembly, for biological processes, cellular components and molecular functions

The complete functional annotation outcomes are available in Additional File 1. Overall, based on the protein sequences extracted from the annotated Hi-C assembly, nearly half of the predicted Gene Ontology (GO) terms (49.18%; 12,265 terms), were classified under the molecular function category, followed by the cellular component (31.68%; 8,133 terms) and biological processes (19.14%; 4,915 terms). As depicted in Fig. 2 some of the most frequent GO biological process terms were ‘translation’, ‘intracellular signal transduction’, ‘carbohydrate metabolic process’, ‘regulation of transcription’ and ‘intracellular protein transport’. The most frequent GO terms in the cellular component category included ‘integral component of membrane’, “nucleus’, ‘cytoplasm’, ‘extracellular region’ and ‘plasma membrane’. In the molecular function group, bindings to nucleic acids and both ATP and GTP, as well as metal ions, including zinc and calcium, were the most common GO terms predicted.

### Genome synteny analysis

**Fig. 3:**
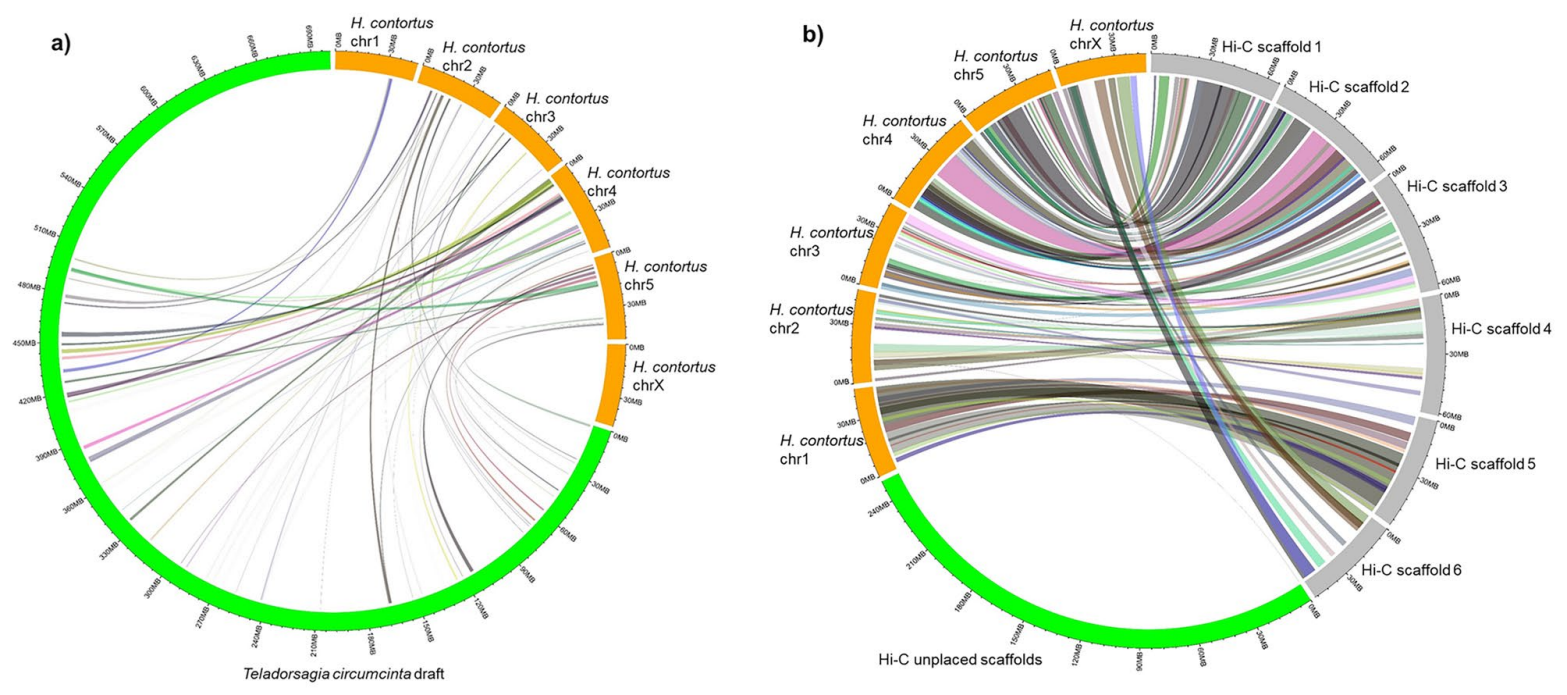
Syntenic relationships between *Haemonchus contortus* genome (orange) and **(a)** the original genome assembly (green) for *T. circumcincta*; and **(b)** the Hi-C genome assembly for *T. circumcincta* (chromosome-length scaffolds in grey; unplaced scaffolds in green). Syntenic links were bundled using the following parameters: --max_gap = 1,000,000 --min_bundle_size = 10,000 min_bundle_membership = 5

Both versions of the *T. circumcincta* assembly were compared with *H. contortus* using pairwise synteny analysis because *H. contortus* has a near-complete genome assembly [11] and, more importantly, phylogenetic analysis shows that it is closely related to *T. circumcincta* [12]. The synteny between the *H. contortus* genome and the original assembly for *T. circumcincta* was relatively poor (Fig. 3A) and greatly improved with the Hi-C assembly (Fig. 3B). It is important to note the strikingly high level of synteny between all six chromosome-length scaffolds in the Hi-C assembly and the six chromosomal sequences of *H. contortus.* Further, synteny analysis allowed identification, for the first time, of the X-chromosome in *T. circumcincta*, with Hi-C scaffold 6 evident as the counterpart of the X-chromosome of *H. contortus*. Interestingly, no syntenic links could be drawn between any unplaced scaffolds in the Hi-C assembly and *H. contortus* genome sequences, perhaps because the parameters were too stringent during the alignment process and when bundling the syntenic links in Circos.

### Orthology analysis

**Fig. 4:**
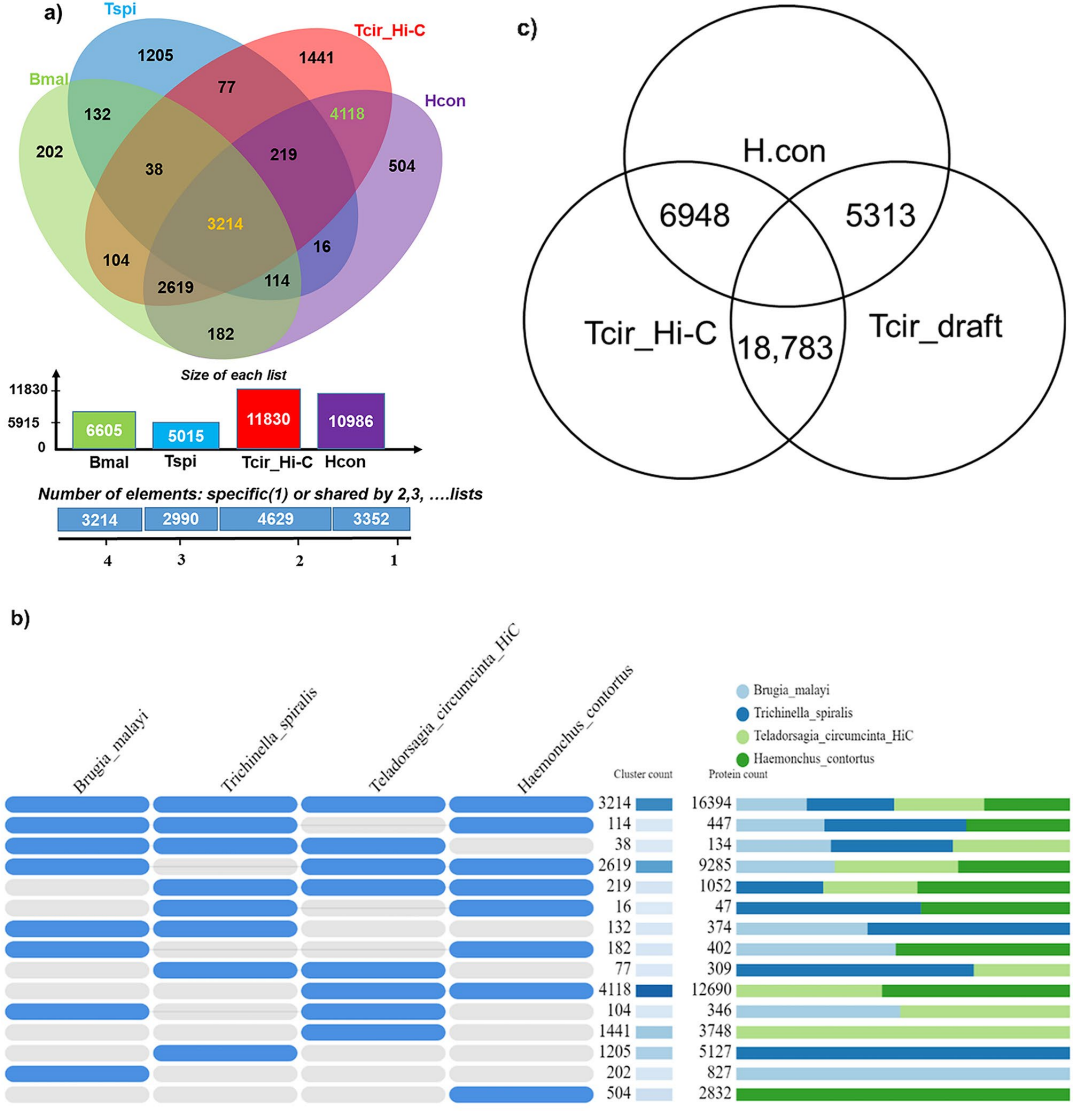
Orthologs shared among helminth species. **(a)** Venn diagram showing comparisons and distribution of orthologous clusters shared among *Burgia malayi* (Bmal, clade-III nematode), *Trichinella spiralis* (Tspi, clade-I nematode), *H. contortus* (Hcon, clade-Va nematode), *T. circumcincta* Hi-C assembly (Tcir_Hi-C, clade-Va nematode) . The species formed 14,185 clusters of which 12,504 were orthologous (contained in at least two species) and 1,681 were single-copy gene clusters. **(b)** Table showing the pattern of occurrence of shared orthologues among Bmal, Tspi, Hcon and Tcir_Hi-C. **(c)** Venn-diagram indicating one-to-one OrthologuesStats inferred from Orthofinder by comparing proteomes of *H. contortus* with *T. circumcincta* Hi-C and *T. circumcincta* draft

Using OrthoVenn2, the protein sequences from annotated *T. circumcincta* Hi-C assembly were also compared with those from *H. contortus*, as well as with two other more distant parasitic nematode species, *Burgia malayi* and *Trichinella spiralis*. Of 12,504 ortholog clusters, 3,214 were shared by all four species (Fig. 4a and b). As expected, the closely related helminths, *T. circumcincta* and *H. contortus*, shared the most orthologs (7,332 clusters), whereas *T. circumcincta* shared only 3,318 orthologs with *B. malayi* and 3,291 orthologs with *T. spiralis*. Using Orthofinder, we also compared the number of orthologs shared between *H. contortus* and the original and Hi-C assemblies of *T. circumcincta*. As shown in Fig. 4c, the Hi-C assembly shared significantly more orthologs (6948) with *H. contortus* than the original draft (5313).

## Discussion and conclusion

The present project aims to improve the current genome reference for *T. circumcincta*, a helminth nematode that is important for small ruminant livestock [8]. By purging alternative haplotypes and using in situ Hi-C to order, orient, correct and anchor draft sequences to chromosomes [10, 13], we have been able to improve the draft genome and create the first chromosome-length assembly for *T. circumcincta*.

The Hi-C assembly is more contiguous and complete than the previously available draft, and, at 614 Mbp, 13% smaller than the original assembly. This reduction in size makes the revised genome of *T. circumcincta* more consistent with that of *H. contortus*, another helminth nematode of the same clade, where the genome size has recently been reduced from 465 Mbp to 283 Mbp [11]. The karyotype (2n = 12) of the *T. circumcincta* genome, identified for the first time in the present analysis, is also consistent with that of *H. contortus* [11], as well as that of *C. elegans*, a model organism that is a free-living nematode [14]. Furthermore, the synteny analysis between the chromosome-length assemblies of *T. circumcincta* and *H. contortus* suggest that chromosomes are syntenic [12] but, while genes are conserved between the two species, the gene order is not, and different regions are linked to different chromosomes [11]. For example, Hi-C Scaffold 6 is syntenic to Chromosome-X on *H. contortus*, whereas Hi-C Scaffold 1 is syntenic to Chromosome 5, Hi-C Scaffold 2 is syntenic to Chromosome 4, and Hi-C Scaffold 3 is syntenic to Chromosome 3.

After genome annotation, there were fewer genes in the Hi-C *T. circumcincta* assembly because haplotypes had been removed and contiguity increased, compared to the original *T. circumcincta* assembly [15]. Although the number of predicted proteins was reduced in the Hi-C assembly, completeness and accuracy were identical for both assemblies, suggesting that, during Hi-C assembly, the rearrangements and reductions in fragmentation increased the number of curated gene models [15]. The single-copy orthologs (SCOs) were also compared across four helminth species from different clades – *T. circumcincta, H. contortus, B. malayi* and *T. spiralis.* As *T. circumcincta* and *H. contortus* belong to the same clade-Va, they share more SCOs (7332) with each other than they share with the other species showing that clade variation can affect the number of shared SCOs within helminths as *T. circumcincta* shares 3318 SCOs with *B. malayi* (clade-III) and 3291 SCOs with *T. spiralis* (clade-I). This variation in shared SCOs is an outcome of speciation and differences among life cycle stages of each helminth – for example, *T. spiralis* with a broad host range, lives in muscle and small intestine [16], whereas infective larvae of *T. circumcincta* and *H. contortus* are found on pastures and infect the abomasum [17], and *B. malayi* requires the mosquito as an intermediate host and infects lymph nodes [18].

Our improved Hi-C assembly still contains several unplaced scaffolds. The analysis of completeness and accuracy of the six Hi-C scaffolds (~ 59% BUSCO; Table 1) suggests that most of the genetic information is retained in the chromosome-length scaffolds. A total of 1275 scaffolds (six chromosome-length scaffolds plus 1269 unplaced scaffolds), has the completeness level like that for the total scaffolds in the Hi-C assembly (52,860), indicating redundancy in the unplaced scaffolds.

In conclusion, our chromosome-length scaffold assembly and annotation have advanced the genomics of the economically important small ruminant nematode parasite, *T. circumcincta* (isolated from Western Australia). The availability of a better reference genome, with greater comprehension of the genetic architecture of Teladorsagiosis, will help phylogenomic analysis of helminths of various clades [19], and help understand the parasite biology and host-parasite interactions. Ultimately, this information should lead to new options for vaccine and drug targets and, most importantly, pave the way to sustainable solutions for gastrointestinal parasitism [20]. Finally, the inclusion of long-read sequencing (from PacBio or Oxford Nanopore) should help resolve the unplaced scaffolds in the current version of the genome assembly [21, 22].

## Materials and methods

### Helminth collection and identification

**Fig. 5:**
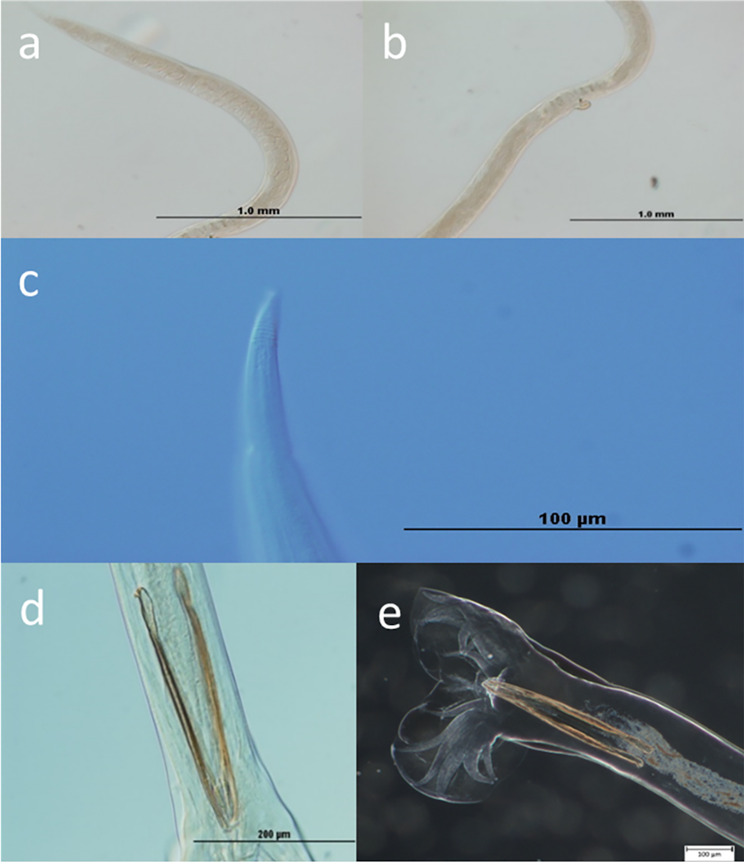
Morphological identification of *T. circumcincta.* (a) Eggs towards the posterior end of the female; (b) Vulvar flap towards the posterior end of the female; (c) Annular rings towards the posterior end of the female; (d) and (e) Spicules towards the posterior, a specific characteristic of the male of this species

Helminths were collected from the abomasum (predilection site for *T. circumcincta)* of sheep obtained from the Western Australian Meat Marketing Company (WAMMCO). The sheep had been naturally infected with *T. circumcincta*, an important helminth in the southwest of Western Australia. The abomasal contents were carefully scraped onto a sieve (mesh size 150 μm) and washed thoroughly and placed in a petri-dish from which individual helminths were removed with the aid of a dissecting microscope. Helminth species were identified based on morphological characteristics (Fig. 5) using differential contrast and compound microscopy. Males were identified by the shape and length of spicules which are up to 450 μm in length; females were identified by the presence of a vulvar flap, annular rings and their body length (10–12 mm; about twice that of males) [3]. Eggs can also be seen in females near the vulvar flap from where they are laid. The worms were then thoroughly washed with physiological saline and stored at − 80 °C until processing. Extracted DNA (see below) was subjected to PCR using helminth specific ITS2 primers, as previously described [23]. Helminth’s identity was confirmed by Sanger sequencing of the PCR product followed by a blastn search against the NCBI database.

### DNA extraction

Briefly, the helminths (100 mature male and female *Teladorsagia circumcincta* in equal ratios) were mechanically homogenized using a sterile micro-pestle in a microcentrifuge tube containing 200 µL of Tris-EDTA buffer, 1% (v/v) β-mercaptoethanol, 200 mg proteinase K, 10 mg/ml RNAase, 0.5 M EDTA and 10% (v/v) sodium dodecyl sulphate. The cell lysate was then incubated at 65 °C for 2 h. After incubation, an equal volume of phenol:chloroform:isoamyl alcohol (25:24:1) was added and the mixture was centrifuged at 10,000 *g* for 5 min. The supernatant was collected into a sterile microcentrifuge tube and resuspended with an equal volume of chloroform:isoamyl alcohol (24:1). After centrifugation, the supernatant was again collected into a sterile microcentrifuge tube, this time with ice-cold ethanol (95% v/v) to precipitate the DNA. The DNA pellet was washed with ethanol (70% v/v) before being resuspended in 50 µL DEPC water. The integrity of the extracted DNA was assessed by electrophoresis on 1% (w/v) agarose gel. The quality and quantity of the DNA were assessed using a NanoDrop 2000 spectrophotometer (Thermofisher, USA) and a Qubit 2.0 fluorometer (Thermofisher, USA).

### PCR amplification of the helminth specific ITS2 region

The ITS2 primer sequences were 5’-CTTAATGATCTCGCCTAGACG-3’ (forward) and 5’-TTTCATCGATACGCGAATCG-3’ (reverse). A 50 µL reaction mixture (reaction buffer 10 µL; forward and reverse primer 2 µL each; DNA polymerase 1 µL; DNA sample 3 µL; water 32 µL) was run through 35 cycles of PCR with MyTaq HS DNA (Bioline, Canada), using following conditions: initial denaturation at 95 °C for 1 min followed by 35 amplification cycles, each comprising denaturation at 95 °C for 15 s, annealing at 54 °C for 30 s, and extension at 72 °C for 10 s.

### Hi-C sequencing, chromosome-length scaffolding and quality assessment

In situ Hi-C sequencing was performed as described previously [10] using 100 adult *T. circumcincta*, including both males and females. We constructed one in situ library which was then sequenced using the Illumina NovaSeq 6000 platform. The generated Hi-C reads were used to anchor, order, orient, and correct misjoins in the existing draft genome assembly (GCA_002352805.1) using the 3D de novo assembly (3D-DNA) pipeline [24]. Before scaffolding with Hi-C reads, the draft assembly was run through purge haplotigs software [25]. The resulting assembly was then polished using the Juicebox Assembly Tools [13]. The resulting contact map was visualized using Juicebox visualization software [13]. QUAST (v5.0.2) was used to assess the assembly metrics [26]. Benchmark for Universal Single Copy Orthologues (BUSCO, v5.1.2) was used in *genome mode* to determine the genome completeness [27]. In this analysis, the sequences for missing BUSCOs in the Hi-C assembly were retrieved manually from https://www.orthodb.org/ (orthoDB v10) and blasted against the draft genome to obtain the relevant scaffolds which were then addedto the Hi-C assembly. The list of added scaffolds can be found in Additional File 2.

### Genome and functional annotations

The original (GCA_002352805.1) and Hi-C integrated draft genome assemblies were annotated using Braker2 v2.1.6 [28]. First, each genome was softmasked using RepeatMasker v4.1.1 [29] with custom repeat library built upon itself by RepeatModeler v2.0.1 [29]. The Braker2 was run with the --etpmode parameter enabled to train GeneMark-ETP [30] with RNA-Seq data and protein hints. The GeneMark-ETP predictions were then used for training AUGUSTUS, following which genes with hints were predicted by AUGUSTUS [30–34]. Five sets of *T. circumcincta* RNA-Seq data (sequence read accession numbers SRX1507697, SRX1507698, SRX2485888, SRX2485887, SRX2485886) derived from two previous studies [8, 35], were downloaded from the NCBI Database and aligned to both the original draft and our improved Hi-C version of genome assemblies, using STAR (v2.7.6a) with default parameters [36, 37]. The *Caenorhabditis elegans* proteome from the UniProt Database served as protein hints when running Braker2. BUSCO was run in *protein mode* to assess the annotation results. After genome annotation, functional analysis was performed using the web-based Gene Ontology Functional Enrichment Annotation Tool (GO FEAT) [38].

### Genome synteny and orthology analyses

Genome-wide synteny was analysed using Cactus v1.3.0 and halSynteny [39] to compare the Hi-C integrated *T. circumcincta* genome assembly with the original GCA_002352805.1 genome assembly, and the genome of a closely related helminth species, *Haemonchus contortus* (GCA_000469685.2). A hierarchical alignment (hal) output file was generated using the Cactus package, and a PSL output file with syntenic links was generated using the halSynteny function within Cactus, using the following parameters: --minBlockSize 10,000 --maxAnchorDistance 1,000,000. The syntenic links were bundled using Circos tools v0.69-8 in Galaxy platform v7 [40, 41] and then visualized using shinyCircos [42]. The single copy orthologs in both the original and Hi-C integrated *T. circumcincta* genome assemblies, as well as the draft assembly of *Haemonchus contortus*, were inferred using Orthofinder [43]. OrthoVenn2 [44] was also used to compare the orthologs between four nematode species: *Burgia malayi*; *Trichinella spiralis*; *H. contortus; T. circumcincta* [12].

## Electronic supplementary material

Below is the link to the electronic supplementary material.

Additional File 1

Additional File 2

## Data Availability

The interactive Hi-C contact map for the genome assembly is available at www.dnazoo.org. The genome assembly and intermediate files can be accessed here; https://www.dropbox.com/sh/czjlxso80stoqts/AAA0wnAO0qttk8i3--rHOPFba?dl=0.
